# The relationship of extent of initial radiological involvement with the need of intensive care, mortality rates, and laboratory parameters in Covid-19

**DOI:** 10.3906/sag-2009-49

**Published:** 2021-06-28

**Authors:** Yusuf AYDEMİR, Yasemin GÜNDÜZ, Mehmet KÖROĞLU, Oğuz KARABAY, Hamad DHEİR, Aysun ŞENGÜL, Selçuk YAYLACI, Havva KOCAYİĞİT, Ali Fuat ERDEM, Özlem AYDEMİR, Ertuğrul GÜÇLÜ, Yusuf YÜRÜMEZ

**Affiliations:** 1 Department of Pulmonology, Faculty of Medicine, Sakarya University, Sakarya Turkey; 2 Department of Radiology, Faculty of Medicine, Sakarya University, Sakarya Turkey; 3 Department of Microbiology, Faculty of Medicine, Sakarya University, Sakarya Turkey; 4 Department of Infectious Diseases, Faculty of Medicine, Sakarya University, Sakarya Turkey; 5 Department of Nephrology, Faculty of Medicine, Sakarya University, Sakarya Turkey; 6 Department of Internal Medicine, Faculty of Medicine, Sakarya University, Sakarya Turkey; 7 Department of Anesthesiology and Reanimation, Faculty of Medicine, Sakarya University, Sakarya Turkey; 8 Department of Anesthesiology and Intensive Care, Faculty of Medicine, Sakarya University, Sakarya Turkey; 9 Department of Emergency Medicine, Faculty of Medicine, Sakarya University, Sakarya Turkey

**Keywords:** Covid-19, extensiveness of radiological lesions, intensive care, mortality rate

## Abstract

**Background/aim:**

It is very important for the efficient use of limited capacity and the success of treatment to predict patients who may need ICU with high mortality rate in the Covid-19 outbreak. In our study, it was aimed to investigate the value of the radiological involvement on initial CT in demonstrating the ICU transfer and mortality rate of patients.

**Materials and methods:**

All PCR-positive patients were included in the study, whose CT, PCR, and laboratory values were obtained simultaneously at the time of first admission. Patients were divided into 4 groups in terms of the extent of radiological lesions. These groups were compared in terms of intensive care transfer needs and Covid-related mortality rates.

**Results:**

A total of 477 patients were included in the study. Ninety of them were group 0 (no lung involvement), 162 were group 1 (mild lesion), 89 were group 2 (moderate lesion), and 136 were group 3 (severe lung involvement). A significant relationship was found between the extensiveness of the radiological lesion on CT and admission to intensive care and mortality rate. As the initial radiological involvement amounts increased, the rate of ICU transfer and mortality increased. The mortality rates of the groups were 0%, 3%, 12.3%, and 12.5%, respectively, and the difference was significant (p < 0.001). Similarly, the ICU transfer rates of the groups were 2.2%, 5.6%, 13.5%, and 17.7%, respectively, and the difference was significant (p < 0.001).

**Conclusion:**

In conclusion, in our study, the strong relationship between the initial radiological extent assessment and the need for intensive care and mortality rates has been demonstrated, and we believe that our results will make a significant contribution to increase the success of the health system in predicting patients who may progress, helping clinicians and managing pandemics.

## 1. Introduction

While the majority of coronavirus disease 2019 (Covid-19) patients recover with asymptomatic or mild symptoms, some of them can progress rapidly to severe respiratory failure and end-organ failure. It is not possible to predict which patients will have a severe course and which patients will need intensive care transfer. We currently have advanced age, comorbid conditions, and some laboratory parameters as predictors of severe disease [1,2]. The most important criteria in determining the need for intensive care transfer are decrease in saturation, radiological progression, and increase in C-reactive protein (CRP), lactate dehydrogenase (LDH), ferritin, and D-dimer values in the follow-up of patients. However, sometimes this condition progresses to an irreversible stage, and patients are lost with macrophage activation syndrome and excessive inflammatory response [3,4]. Therefore, before these laboratory parameters and saturation values deteriorate, it is very valuable to predict patients who may progress at an earlier stage. In addition, intensive care capacities have been exceeded considerably in many countries during the pandemic and this continues to be a problem in triage management. Any method that may help predict which patients are associated with a higher mortality and require ICU care may improve patient care and utilization of the capacity [3]. It may also result in improved patient outcomes by providing early access to advanced supportive care, such as high flow oxygen therapy, noninvasive mechanical ventilators, and antiinflammatory and anticytokine therapy.

Covid-19 frequently involves the lungs and causes viral pneumonia. Computerized tomography (CT) of the chest is quite sensitive in identifying viral pneumonia [5]. As the sensitivity of the real-time reverse transcriptase polymerase chain reaction (PCR) method, the gold standard in diagnosing Covid-19, falls short of expectations, chest CT scans have been extensively used for the early diagnosis and in treatment decisions [6,7].

The aim of this study was to investigate the value of thoracic computed tomography performed simultaneously with PCR positivity and at the time of initial presentation in predicting disease progression and ICU transfer rate. For this purpose, in patients with definite diagnosis of Covid-19, extent of initial radiological involvement degree was grouped (as no lesion, mild, moderate, and severe involvement) and the relationship between the groups and certain laboratory parameters, ICU transfer, and mortality rates was examined.

## 2. Materials and methods 

This study was conducted retrospectively between 14 March and 4 May in the 3rd stage Education and Research Hospital, which is the only pandemic center in a city with a population of 1 million. Only patients who had a CT scan and certain laboratory examinations on the same day with Covid-19 specific PCR positivity were included in the study. (All of the PCR, CT, and laboratory parameters used in the study belong to the first admission day). Thus, it was investigated whether the data obtained on the first day could be a marker for the progression of the disease in the future.

Patients who were PCR-negative but clinically Covid-19–compatible, and those with different PCR positivity and CT/blood analysis days were excluded from the study. Additionally, since alanine aminotransferase (ALT), creatine kinase (CK), CRP, LDH, lactate, D-dimer, ferritin, erythrocyte sedimentation rate (ESR), white blood cell (WBC), hemoglobulin (Hg), lymphocyte, and platelet (PLT) values are evaluated for study, patients with hematological disorders, active liver disease, or malignancies that could potentially adversely affect the values of the determined blood parameters were excluded.

**Figure 1:**
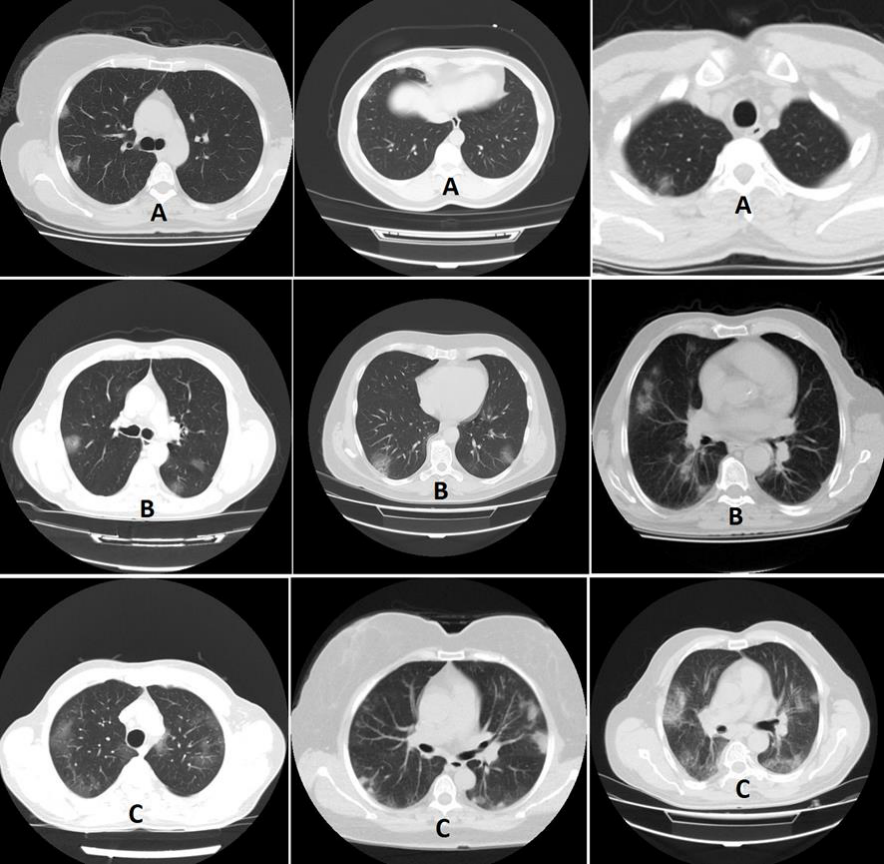
A: Group 1 patients, unilateral lesions limited to a minimal segment or lobe. B: Group 2 patients, including several lobes or segments but fewer than 3 lesions. C: Group 3 patients, widespread bilateral lesions.

Thoracic CT images taken on the same day with PCR were evaluated by the same radiologist to eliminate interobserver differences. The chest CT scans were reviewed by the experienced and blind radiologist for signs of viral pneumonia, including ground-glass opacities, consolidation, and presence of nodules. In addition, lung segments with lesions were detected. Patients were categorized according to the extent of the lesions. Patients with no lesions associated with viral pneumonia were assigned to group 0, those with unilateral and minimal lesions confined to one segment or lobe were assigned to group 1 (Figure 1A), patients with less than 3 lesions that involve multiple lobes or segments were assigned to group 2 (Figure 1B), and patients with diffuse and bilateral lesions were assigned to group 3 (Figure 1C). Sample images of radiographic findings for each group are shown in Figure 1.

All patients included in the study were followed for 2 months. During this period, patients whose general condition worsened and who were transferred to intensive care and died due to Covid-19, despite the standard treatment and care performed according to the national guideline were recorded. ICU transfer rates, mortality rates, and the laboratory values on the first admission day of the four groups were compared. Thus, the relationship between the prevalence and extensiveness of Covid-19 lesions in thorax CT at the time of admission and Covid-19 progression was evaluated.

### 2.1. Statistical analysis

Descriptive statistics were presented as numbers and percentages for categorical variables and as standard deviations, minimum and median value for numerical data. Chi-square test was used to compare paired groups containing categorical data. Normality was evaluated with the Shapiro–Wilk test. The Mann–Whitney U test was used for numerical variables that did not show normal distribution. The Kruskal–Wallis test was utilized to compare the numerical data of three or more nonnormally distributed independent groups. p < 0.05 was considered statistically significant. SPSS v 20.0 (IBM SPSS Statistics for Windows, Version 20.0; Armonk, NY, USA) package program was used for analyses.

## 3. Results

The 477 patients with a PCR-confirmed diagnosis were included in this study. The mean patient age was 49.2 years, and 262 (54.9%) patients were male. Fifty-seven of the patients (11.9%) were asymptomatic. The average time until the first admissions was 1.43 days, and 387 patients had lesions consistent with Covid-19 on their chest CT scans (81.1%). Forty-seven patients (9.8%) were admitted to the ICU. Thirty-three patients (6.9%) died. Patient demographics, symptom durations, laboratory test results, and radiographic findings are shown in Table 1.

**Table 1:** Demographic data’s clinical, radiological, and laboratory values in patients.

	n	%				
Total	477					
ICU –/+	47/430	9.85				
Exitus –/+	33/444	6.91				
Sex: male/female	262 / 215	54.9/45.1				
Symptom duration		No symptom	1 day	2 day	3 day	4 day
n (%)		57 (11.9)	213 (44.7)	156 (32.7)	48 (10.1)	3 (0.6)
Radiological findings						
0 (no lesion)	90	18.9				
1 (mild)	162	34				
2 (moderate)	89	18.7				
3 (severe)	136	28.5				
	n	NV	mean	± SD	median	min- max
Age			49.2	17.8	48	0-92
Symptom duration			1.43	0.85	1	0-4
Laboratory findings						
ALT	477	0–50 (U/L)	32.3	27.7	24	3–308
CK	439	0–171 U/L	149.1	297.6	83	8–4001
LDH	454	0–248 (U/L)	261.7	110.3	228	119–1161
CRP	473	0–5 (mg/L)	36	55.4	13.6	0.3–386
Lactate	405	0.5–1.6(mmol/L)	1.7	0.7	1.6	0.5–5.8
D-dimer	449	0–500 (ug/L)	868.7	2684	291	2–34200
Ferritin	447	21–274 (mcg/L)	309.9	479	160	3–4096
Sedimentation	95	5–20 (mm/h)	28.7	26.9	17	2–98
WBC	477	5.6–10.2 (K/uL)	6.26	2.4	5.7	2.3–22
Hemoglobuline	477	12.2–18.1(g/dL)	13.5	1.5	13.7	7.8–17
PLT	477	142–424 (K/uL)	195.3	69.4	183	22–663
Lymphocyte	477	0.6–3.4 (K/uL)	1.60	0.76	1.48	0.25–5.36

There was a significant correlation between the extent of CT involvement and ICU admission. There were 90 patients in group 0 (without radiographic involvement). Only 2 of them (2.2%) had been transferred to ICU. Nine patients (5.6%) in group 1 (mild involvement), 12 patients (13.5%) in group 2 (moderate involvement), and 24 patients (17.7) in group 3 (severe involvement) were transferred to ICU.

**Figure 2:**
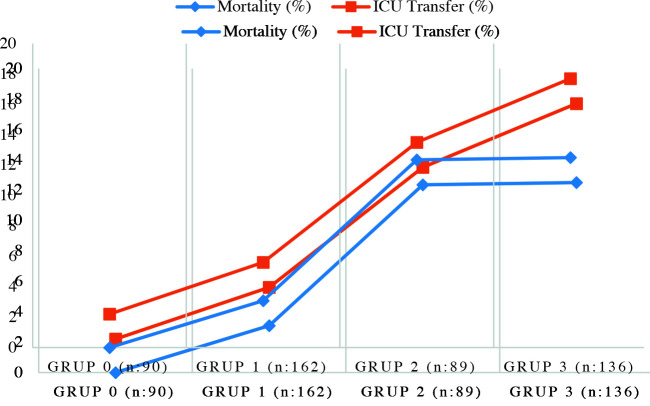
Mortality and ICU transfer rates according to radiological groups.

There was a significant correlation between the extent of lung involvement and ICU admission (p < 0.001). The results are shown in Table 2. The rate of ICU admission increased in parallel to the extent of lesions on radiographic images (Figure 2).

**Table 2:** Relationship between the extent of lesions on radiographic images and need for ICU.

	No needfor ICU	Need forICU (%)	p-value
CT lesion			
Absent n: 90	88	2 (2.2)	0.005*
Present n: 387	342	45 (11.6)	
Extensiveness of CT lesions			
0 (no lesion) n: 90	88	2 (2.2)	<0.001**
1 (mild) n: 162	153	9 (5.6)	
2 (moderate) n: 89	77	12 (13.5)	
3 (severe) n: 136	112	24 (17.7)	

*Chi-square test was used, ** The Shapiro–Wilk test was used for normality and the Kruskal–Wallis Test was used for comparison of groups.

There was a significant correlation between the extent of CT involvement and mortality. While none of the 90 patients with no imaging findings died, all the 33 patients who died had radiographic involvement. Moreover, we found a significant correlation between the extent of radiographic involvement and mortality. Patients with more extensive radiographic lesions had a higher mortality rate. The mortality rates were 0% for group 0 (no lesion), 3.8% for group 1 (mild), 12.3% for group 2 (moderate), and 12.5% for group 3 (severe). The difference was significant (p < 0.001). The results are shown in Table 3 and Figure 2.

**Table 3:** Relationship between the extent of lesions on radiographic images and mortality.

	Survival	Nonsurvival (%)	p-value
CT lesion			
Absent n: 90	90	-	0.001*
Present n: 387	354	33 (8.5)	
Extensiveness of CT lesions			
0 (no lesion) n: 90	90	-	<0.001**
1 (mild) n: 162	157	5 (3.08)	
2 (moderate) n: 89	78	11 (12.35)	
3 (severe) n: 136	119	17 (12.5)	

*Chi-square test was used, **The Shapiro–Wilk test was used for normality and the Kruskal–Wallis Test was used for comparison of groups.

**Figure 3:**
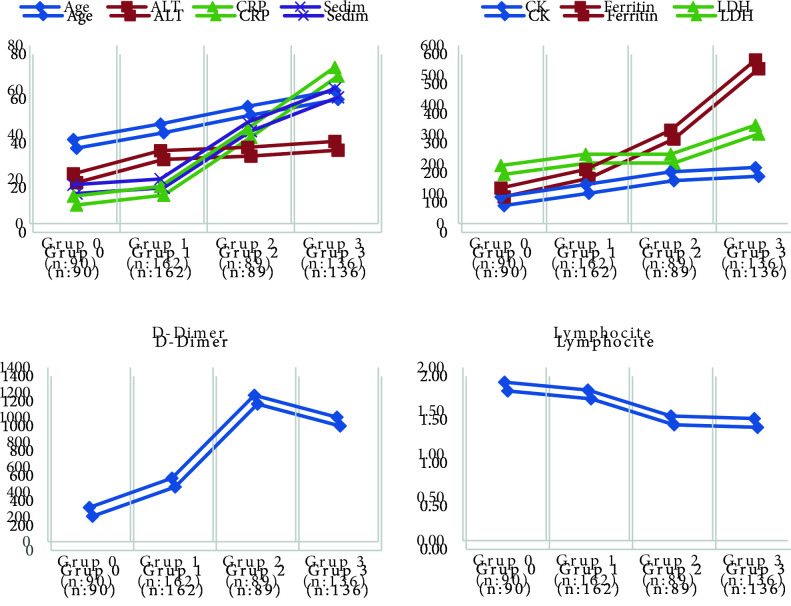
Laboratory values according to radiological groups.

The groups were also compared in terms of laboratory parameters. D-dimer, ferritin, LDH, CRP, ALT, CK, and sedimentation were significantly higher, and lymphocyte values were significantly lower in group 2 and group 3. The results are shown in Table 4. As the degree of lung involvement increased, LDH, CRP, D-dimer, and ferritin values also increased, and lymphocyte values also decreased (Figure 3). There was no significant difference in lactate, WBC, and Hg levels. 

**Table 4:** Comparison of laboratory parameters according to radiological groups.

	GROUP 0No pneumonia group n: 90	GROUP 1Mild pneumonia group n: 162	GROUP 2Moderate pneumonia group n: 89	GROUP 3Severe pneumonia group n: 136	p-value0 vs 3	p-value1vs 3	p-value2 vs 3	p-value0 vs 1
Age	37.86	44.76	52.66	59.72	<0.001	<0.001	0.001	<0.001
ALT	22.36	32.81	34.31	36.93	<0.001	0.03	0.544	<0.001
CK	90.43	132.01	174.16	189.23	<0.001	0.019	0.292	0.081
LDH	195.8	234.06	233.7	331.85	<0.001	<0.001	<0.001	<0.001
CRP	12.32	16.78	42.73	70.25	<0.001	<0.001	<0.001	<0.001
D-dimer	274.3	510.5	1176.5	1000.9	<0.001	<0.001	0.069	0.013
Ferritin	120.1	182.8	315	551.4	<0.001	<0.001	<0.001	0.002
Sedim	17.45	20.1	45.47	60.69	0.001	0.003	0.16	0.038
Lymph	1.83	1.74	1.44	1.41	<0.001	<0.001	0.367	0.292

The Shapiro–Wilk test was used for normality and the Mann–Whitney U test was used for comparison of groups.

## 4. Discussion 

Predicting the patients of who will require ICU admission and have higher mortality rates are of major importance in the battle against Covid-19. Because, during the pandemic, appropriately deploying health care resources and optimizing the utilization of the limited number of ICU beds and medical devices, such as ventilators, has become critical for the successful management of this condition. For this purpose, many studies have been conducted investigating the prognostic factors of severe disease and mortality in Covid-19. As a result of these studies for mortality associated with Covid-19; age, sex (male), comorbidities (such as obesity, cardiovascular disease, diabetes, hypertension, COPD, malignancy, and chronic kidney disease), height of inflammatory markers such as interleukin-6, CRP, and ESR, higher blood coagulation measurements such as D-dimer and ferritin, liver and renal function disorders such as bilirubin, ALT, and blood urea nitrogen elevation, higher WBC count and lower absolute lymphocyte count were defined as prognostic factors [8,9].

We know that, during the follow-up of the patients, impairment in the above-mentioned laboratory tests and radiological progression indicate that the disease is getting worse. Beyond what is known, in our study, unlike other studies, we investigated the relationship between the extent of lung involvement not during the follow-up of the patients, but during the first admission CT scans, and mortality and progression rate. In our study, we found that scoring the thorax CT taken on the first day of admission is quite important in showing mortality and intensive care transfer rate. There was a significant correlation between the extent of radiographic involvement and mortality rate. All 33 patients who died had moderate or severe lung involvement findings on their CT scans and all patients in the group 0 (no lesions) survived. As the extent and number of lesions increased, mortality rates increased significantly. We also found a significant relationship with the ICU transfer rate. Similarly, 45 of 47 patients with severe disease progression requiring ICU admission had lung involvement at first admission day. As the size and number of the lesions increased, the ICU transfer rates increased significantly. The results of this study suggest that patients with extensive lung involvement at baseline may need close clinical monitoring and admission to the intensive care unit and have a higher mortality rate.

In this study, all patients who met the diagnostic criteria for a potential case were tested with PCR and had a chest CT scan performed at the baseline. Among 477 consecutive patients who tested positive with PCR, 287 had lesions suggestive of Covid-19 on their CT scans. The pulmonary involvement rate in our cohort was 81%. Among patients with lesions on radiographic images, 42% had mild, 13% had moderate, and 35% had severe lung involvement. 

The clinical presentation of patients with Covid-19 varies broadly from asymptomatic cases to patients with severe respiratory failure who need mechanical ventilatory support. The gold standard in the diagnosis of this condition is demonstrating viral RNA using nucleic acid amplification tests (NAAT). The sensitivity of NAAT used to diagnose Covid-19, however, also falls short of meeting expectations. Only 60% of potential Covid-19 patients with clinical, radiographic, and laboratory findings test positive with PCR [6,10]. A significant proportion of patients who repeatedly test negative with PCR have positive antibody test results later on, which reinforces the perception that PCR test is not reliable in the diagnosis of Covid-19 [10,11].

This shortcoming of the PCR test has resulted in the common use of chest CT scans in the diagnosis of Covid-19 and when deciding to start the treatment for it. Based on the literature reports, chest CT has a diagnostic value of more than 90% and is more reliable than a PCR test. In the great majority of moderate-to-severe Covid-19 patients who require hospitalization, the most significant presentation is pneumonia. The typical chest CT findings for Covid-19 include ground-glass opacities, consolidation, and cobblestone appearance. In a 919-patient series reported by Sana et al., 88% of the patients had ground-glass appearance and 31% had consolidation. Lesion distribution was reported as bilateral in 87%, multilobar in 79%, and peripheral in 76% of the cases [7]. Typical imaging findings of Covid-19 have been well-described, which improves the diagnostic value of the CT scan [12]. In a cohort of 1014 patients, the correlation between CT scans and PCR test was investigated, and the sensitivity of the CT was reported as 97%. Based on these reports, chest CT is commonly and reliably used in the diagnosis of Covid-19 and when deciding to introduce treatment for it. In addition to the role of chest CT in the diagnosis and treatment decisions, this study demonstrates that a simple scoring system may help to predict the disease progression.

Based on estimates from three major cohorts, 30%–40% of Covid-19 patients are asymptomatic. In the cruise ship Diamond Princess, 41% of the patients were asymptomatic at baseline and remained free of symptoms for the entire duration of the observation period. Imaging findings were observed in 52% of the asymptomatic cases, and 7% of those who progressed to a disease severity which required oxygen support. Based on reports from a study that reviewed 3200 Covid-19 patients who died in Italy, 5.7% of the patients were asymptomatic when they first presented to the hospital. The available data suggests that clinical symptoms at baseline fall short of predicting progression and mortality in severe disease. In previous reviews, a clear relationship was not found between death in Covid-19 patients and the presence of clinical symptoms [8,9]. 

The extent of radiographic involvement may prove to be useful in this situation.

In this study, we also evaluated the correlation between the extent of radiographic involvement and certain laboratory parameters. A standard test panel was created based on available Covid-19 data and a significant correlation was determined between the D-dimer, ferritin, LDH, CRP, ALT, CK, lymphocyte, and sedimentation at baseline and the extent of radiographic involvement. In addition to the presence of radiographic involvement, the extent of the involvement also showed a strong correlation. LDH had the strongest correlation with the extent of lung involvement, with LDH levels increasing in parallel to the extent of involvement and distinct differences were observed between the four patient groups. D-dimer, CRP, and ferritin levels also increased in proportion to the extent of lesions and significant differences were identified in three of the four groups. In addition to the correlation between increased levels of ferritin, D-dimer, CRP, and low lymphocyte counts and ICU admission and mortality reported by previous studies, this study showed that the extent of radiographic involvement is also related to laboratory values.

This result further reinforces the importance of the extent of radiographic involvement in first admission. The key strength of this study is that all radiographic and laboratory tests were performed concurrently at baseline. It is a known fact that D-dimer, CRP, and ferritin levels increase and lymphocyte count decreases as the disease progresses and there is also radiographic progression. Our study was built on whether the radiological size and number score we obtained on the first day could predict the progression of patients, their going to ICU, and mortality. Our results suggest that based on data obtained at initial admission presentation, it is possible to predict which patients may progress.

The strength of our study is that it is the first study on this area, as far as we know. Our study has some limitations. To what extent accounting for these radiological factors will improve clinically important outcomes is a question that cannot be addressed with our results. In addition, there are multiple individual prognostic factors for outcome prediction and this is a limitation of our study. Multivariable models provide a solution to this limitation; our work can also provide solid grounds for development of these prognostic tools. For example, they could select a set of prognostic factors for which there is high certainty in a significant risk incremental increase (e.g., age, sex, comorbidities, respiratory failure, impaired certain laboratory tests, and extent of radiological involvement) and use them to define hospitalization rules for patients consulting to the emergency department. The second limitation of this study is that patient symptoms could not be included in the evaluation because the records were found to be unreliable. Thus, the correlation between the extent of radiographic involvement and patient symptoms could not be evaluated. However, the time elapsed between the onset of symptoms and the first presentation was evaluated because patients’ first admission to the hospital and the onset of symptoms may be different for each patient, and this difference is important in terms of disease stage. Radiological involvement can change in every stage of the disease. In our study, the average duration of symptoms was 1.43 days, and 89% of the patients applied within the first 2 days. The shortness of this period can be explained as the very high awareness in the society because our study was conducted in the very early stages of the pandemic. Therefore, there were no late-stage patients in our patient population, and this did not affect our results.

In conclusion, we were able to demonstrate a strong correlation between the extent of radiographic involvement at baseline and the need for ICU admission, mortality rates, and laboratory parameters. Our results may be helpful in predicting which patients are at greater risk of disease progression and thus may significantly contribute to improving the success of the health care system in managing this pandemic. In this way, patients who can be transferred to the intensive care unit can be followed more carefully and more closely. For example, some wards can be designed as first-stage intensive care with supplement of monitors and patients who are likely to progress can be followed in these departments.

In this respect, we believe that the results of our study can benefit enabling patients to access advanced treatments earlier and ICU bed management.

## Ethical approval

The present study protocol was conducted after approval of the Ethics Committee of Sakarya University Faculty of Medicine (No:71522473/050.01.04/142) and approval of the National Ministry of Health (No: 2020-05-02T16_41_11). This study was conducted in accordance with the principles of the Declaration of Helsinki.

## Informed consent

This study was conducted with Sakarya University Medical Faculty Ethics Committee (No: 71522473 / 050.01.04 / 142) and with the permission of the Ministry of Health.
